# Vasopressors and Nutrition Therapy: Safe Dose for the Outset of Enteral Nutrition?

**DOI:** 10.1155/2020/1095693

**Published:** 2020-02-10

**Authors:** Luís Henrique Simo˜es Covello, Marcella Giovana Gava-Brandolis, Melina Gouveia Castro, Martins Fidelis Dos Santos Netos, William Manzanares, Diogo Oliveira Toledo

**Affiliations:** ^1^Department of Critical Care of Barretos Cancer Hospital, Barretos, Brazil; ^2^Department of Research of Hospital Sa˜o Luis Itaim, Sa˜o Paulo, Brazil; ^3^Department of Education of Hospital Israelita Albert Einstein, Sa˜o Paulo, Brazil; ^4^Department of Science Information of Barretos Cancer Hospital, Barretos, Brazil; ^5^Department of Critical Care, University Hospital of Universidad de la Republica (UDELAR), Montevideo, Uruguay; ^6^Department of Critical Care of Hospital Israelita Albert Einstein, Sa˜o Paulo, Brazil

## Abstract

**Background and Aims:**

Patients with hemodynamic instability need to receive intensive treatment as fluid replacement and vasoactive drugs. In the meantime, it is supposed to initiate nutritional therapy within 24 to 48 hours after admission to the intensive care unit (ICU), as an essential part of patient's intensive care and better outcomes. However, there are many controversies tangential to the prescription of enteral nutrition (EN) concomitant to the use of vasopressor and its doses. In this way, the present study aimed to identify what the literature presents of evidence to guide the clinical practice concerning the safe dose of vasopressors for the initiation of nutritional therapy in critically ill patients.

**Methods:**

This review was carried out in PubMed, ProQuest, Web of Science, and Medline databases. The descriptors were used to perform the search strategy: Critical Care, Intensive Care Units, Vasoconstrictor Agents, and Enteral Nutrition. Inclusion criteria were patients of both genders, over 18 years of age, using vasoactive drugs, with the possibility of receiving EN therapy, and articles written in English, Portuguese, and Spanish. In addition, exclusion criteria were case reports, non-papers, and repeated papers.

**Results:**

10 articles met our inclusion criteria.

**Conclusion:**

It was observed that there are many controversies about the supply of EN in critically ill patients using vasopressor, especially about the safe dose, and it was not possible to identify a cutoff value for the beginning therapy. Despite the drug doses, clinical signs are still the most important parameters in the evaluation of EN tolerance.

## 1. Introduction

Critically ill patients are often hemodynamically unstable (or at risk of becoming unstable) owing to hypovolemia, cardiac dysfunction, or alterations of vasomotor function, leading to organ dysfunction, deterioration into multiorgan failure, and eventually death [1].

Under resting and normal conditions, around 25% of cardiac output is located in the splanchnic circulation. Shock is characterized by blood flow redistribution with vasoconstriction at splanchnic circulatory level and in peripheral tissues, in an attempt to maintain vital organs perfusion. This can give rise to an imbalance in the oxygen supply/demand ratio at intestinal level, with resulting ischemia [2]. The reported incidence ranges between 0.3% and 8.5%, with mortality ranging from 46% to 100% [3].

Patients with hemodynamic instability need to receive rapid and intensive treatment to restore homeostasis. Therapeutic measures may include fluid resuscitation, vasopressors, or inotropic agents. These drugs are used to raise blood pressure in order to adapt to perfusion of organs and tissue [4].

The term vasoactive drug is used for drugs that have vascular peripheral, pulmonary, and cardiac effects, whether it is directly or indirectly. These drugs act on receptors located in the vascular endothelium and their effects are dose-dependent [4].

Enteral Nutrition Therapy (ENT) is part of the essential care of patients in the intensive care unit (ICU) [5]. It is recommended, due to several studies that demonstrate better outcomes [6], that nutritional support should be started 24 to 48 hours after admission to the ICU, since the patient has been resuscitated and with hemodynamic stability [7].

ENT, when applied properly and early at the correct time, reduces the incidence of unfavorable outcomes in critically ill patients as well as the risk of infectious complications and ICU length of stay [8].

Meanwhile, in the context of the critical patient, there are many controversies tangential to the prescription of ENT, especially when there is a concomitant need for vasopressor support. It is important to emphasize that the use of vasopressor per se does not impair enteral nutrition [9]. However, the use of vasoactive drugs has systemic effects that increase the risk of intolerance, among other complications, such as nonocclusive intestinal ischemia.

Currently, consensus among experts determines hemodynamic instability as a contraindication to any nutritional strategy, either enteral or parenteral. On the other hand, once clinical stabilization is achieved (from a macro/microhemodynamic) NT can be instituted, preferably by enteral route [10].

The aim of this study was to identify the current evidence to guide the clinical practice regarding the safe dose of vasopressor for the beginning of NT in ICU patients.

## 2. Materials and Methods

A literature review of articles was conducted, seeking the existence in the literature of evidence that guided the safe dose of vasopressors for the beginning of ENT.

PubMed, ProQuest, Web of Science (WOS), and Medline were used as database. The following descriptors were searched for: Critical Care, Intensive Care, Intensive Care Unit, Vasoconstrictor Agents, Vasoconstrictors, Enteral Nutrition, and Enteral Nutrition. Vasoactive drugs were also used as free terms. The descriptors were validated in DecS (descriptors in health science) and MeSH (medical subject headings).

Inclusion criteria were applied: patients of both genders, over 18 years of age, using vasoactive drugs, and having the possibility of receiving EN. Moreover, regardless of year of publication, our search was not restricted to articles written in English and therefore we included articles in Portuguese and Spanish. Articles were excluded if they were case reports, non-articles (posters, oral presentations, and annals of congresses), or repeated papers.

## 3. Results

**Figure 1:**
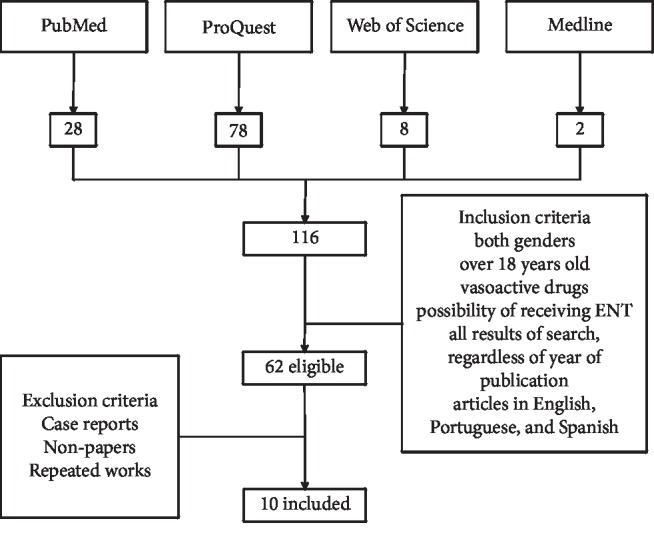
Articles selected with search strategy.

From the search strategy, there were 116 articles. After the initial evaluation 54 studies were withdrawn because they did not meet the inclusion criteria and 52 were withdrawn in the evaluation of the exclusion criteria, 7 of which were repeated, 1 was case report, and 44 were non-articles; therefore 10 articles were included (Figure 1). The main findings are shown in Table 1 and will be discussed forward.

## 4. Discussion

Nutrition support is the key to sustaining life. Currently, the evidence points to the early initiation of nutrition therapy in critically ill patients, that is, up to 48 hours after admission to the ICU, provided that they are adequately resuscitated in relation to blood volume and with hemodynamic stability [6, 7, 11, 12].

In 2016 A.S.P.E.N. recommended not feeding patients who have blood pressure lower than 50 mmHg and patients who need to initiate or increase vasoactive drugs [13].

Furthermore, in Canada, the critical care nutrition group question the obligatory hemodynamic stability to initiate EN, as there is enough evidence about the benefits of early EN in ICU patients. In fact, according to current literature, EN have demonstrated maintenance of mucosal integrity of TGI, decreased bacterial translocation, increased splanchnic blood flow, improved wound healing, improved immune function, and modulated response to tissue damage [6, 7, 11, 14]. In addition, in endotoxic and septic shock models, enteral feeding improved hepatic artery and portal vein blood flow, superior mesenteric artery blood flow, intestinal mucosal microcirculatory flow, hepatic microcirculatory flow, hepatic and intestinal tissue oxygenation, and hepatic energy stores [15–17]. Added to these benefits, better clinical outcomes were observed, such as reducing the severity of illness, complications, and ICU LOS [11].

Despite the benefits mentioned above, a justification pointed out by many professionals not to initiate early EN is the possibility of intestinal ischemia. Presence of abdominal distension, pain disproportionate to physical examination of the abdomen, high nasogastric output, metabolic acidosis without obvious cause, and digestive hemorrhage may be indicative of this complication, although there are no specific clinical signs or markers for early diagnosis [3, 18]. It is often accompanied by hypotension and hypovolemic shock [14]. And some radiographic signs could appear like dilated thickened loops of bowel with thumbprinting, air in the wall of gastrointestinal tract, portal venous gas, and air in the peritoneal space. In this scene, ENT should be discontinued upon worsening of hemodynamic instability or systemic inflammatory response, followed by reevaluation of GI perfusion [19].

**Figure 2:**
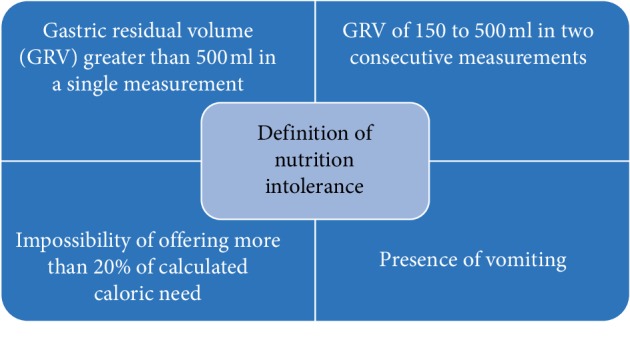
Definition of nutrition intolerance commonly used in the literature.

The present evidence does not attest that nutrition by the GIT is responsible for ischemia [19, 20]. Besides that, the most common and well documented complication related to vasopressor use is EN intolerance [13], which occurs in 30 to 70% of patients and may be caused by altered intestinal perfusion [21–24]. Intolerance to nutritional therapy implies a higher risk of aspiration pneumonia, longer ICU LOS, and increased ICU mortality [25]. In Figure 2 different definitions of nutrition intolerance are shown.

In a very elegant multicenter study including ICU patients on mechanical ventilation, institution of EN in hemodynamically unstable patients led to higher rates of vomiting (34%), diarrhea (36%), intestinal ischemia (2%), and colonic pseudoobstruction (1%). Therefore, the authors concluded that the use of the intestinal tract in shocked patients with clear signs of hypoperfusion seems to actually increase the incidence of intestinal ischemia [12], indicating that EN should be initiated only after the achievement of macro- and microhemodynamic stability.

Regarding the dose of vasoactive drug, studies have found different outcomes. Doses around 0.14 *μ*g/kg/min were shown for patients who tolerated the diet, including doses of ≤12.5 mcg/min of norepinephrine, although another study presented a dose of 0.25 *μ*g/kg/min for patients who did not tolerate diet [3,7,26].

Furthermore, a review made by Allen found that doses around 3–10 *μ*g/kg/min of dopamine, 12 *μ*g/kg/min of dobutamine, and 6–25 *μ*g/min of norepinephrine are safe to EN tolerance [14].

In addition, studies have correlated that increasing doses of vasoactive drugs are related to some degree of intestinal lesion [3, 19]. However, several studies have shown that the use of vasopressors did not worsen and even may be able to improve intestinal perfusion, in doses like 0.2 *μ*g/kg/min to 5 *μ*g/kg/min of dopamine, 0.3 *μ*g/kg/min of epinephrine, 0.9 *μ*g/kg/min of norepinephrine, and 0.5 g/kg/min of phenylephrine [13, 14, 26].

It seems that stable and low doses of vasopressors have better outcomes and should be withheld in patients who are hypotensive (mean arterial blood pressure (PAM) <50 mmHg), as well as in those patients for whom catecholamines are being initiated, or in patients for whom escalating doses are required to maintain hemodynamic stability [8].

Specific effects of vasoactive drugs on the GI tract are mixed. The effect of norepinephrine varies with the underlying pathophysiology associated with the hemodynamic instability. As an example, it is well known that, in those patients with sepsis/septic shock, norepinephrine, epinephrine, and phenylephrine may be able to increase MAP and the GI blood flow. However, these drugs also caused a decrease in intestinal blood flow and the overall fraction of cardiac output to the GI tract [27, 28]. Indeed, there are also evidence that the use of inotropes does not directly interfere with intestinal BF [13].

So far, few studies indicate that vasopressin decreases mesenteric and splanchnic blood flow. Results from studies in humans reported that vasopressin was responsible for this decreased flow even in the presence of additional catecholamines such as norepinephrine [13, 29–31]. Additionally, another interesting study demonstrated that addition of vasopressin resulted in decreased EN tolerability [26].

In general, dopamine, epinephrine, and vasopressin negatively regulate GI blood flow, which is minimally affected by norepinephrine. Nonetheless, inotropes such as dobutamine and milrinone, when used alone, increase cardiac index and GI blood flow [13]. A summary of main pharmacological and hemodynamics effects of vasopressors on GIT is shown in Table 2. In clinical practice, multidrug combinations, individual drug susceptibility variations, and dose-dependent effects of vasoactive agents are common phenomena. In this scenario, the effects of vasopressors on the GIT and splanchnic blood flow would appear to be dose related, but this has not been fully investigated so far. Therefore, based on current evidence, it is really hard to estimate the risk of intestinal ischemia when combining different types of vasoactive drugs in ICU patients [14, 19].

Yang brought important points in his study as a consensus: the degree of risk for NOBN is difficult to determine based solely on the absolute dose of vasoactive agents. Early EN should start when the patient is on stable or declining doses of vasopressors. Trophic feeding or nutrition (10–20 mL/h) with step-up progression is probably the best strategy for this patient population. Any radiographic or laboratory monitoring cannot take the place of tight observation of clinical symptoms and complaints. Daily and careful monitoring of the possible alarm signs of intestinal ischemia in these high-risk patients is of crucial importance. Clinical signs such as increased gastric residue, a rise in intra-abdominal pressure to over 15 mmHg (particularly when associated with recent oliguria), or sudden worsening of the hemodynamic situation of the patient should be regarded as possible indicators of intestinal ischemia (2). The casual relation between EEN and NOBN has yet to be clearly established in hemodynamic instability [19].

Thus, existing studies on the subject differ on the best time to initiate nutrition in critically ill patients receiving vasoactive drugs in relation to their dosages and should take into account blood volume, blood pressure, hemodynamic stability, possibility of intestinal ischemia, in addition to the maintenance of the mucosal integrity of the GIT and the reduction of the severity of the diseases and eventual complications, as well as the reduction of the length of ICU stay. Bruns suggests that most postoperative patients requiring vasopressor therapy can be safely initiated and advanced on enteral nutrition [3].

According to the main NT guidelines in severe patients, EN support should not be initiated during hemodynamic instability, or with increasing doses of vasopressor. There is a significant risk of serious complications, such as intolerance and nonocclusive intestinal ischemia. It should be emphasized that the use of vasopressor per se does not contraindicate the institution of nutritional therapy; meanwhile ENT may be considered with caution in patients undergoing withdrawal of vasopressor support. The literature does not indicate a specific cutoff point to the value of these agents to contraindicate or suspend ENT and there is not even a consensus among intensivists at a cutoff point of vasopressor dose that determines whether it is a high or a low dose [8, 27].

If hemodynamic resuscitation is established in conjunction with the correction of hydroelectrolytic disorders, NT can be instituted as early as possible, as current evidence suggests that this timely intervention has a positive impact on relevant clinical outcomes in seriously ill patients [32].

**Figure 3:**
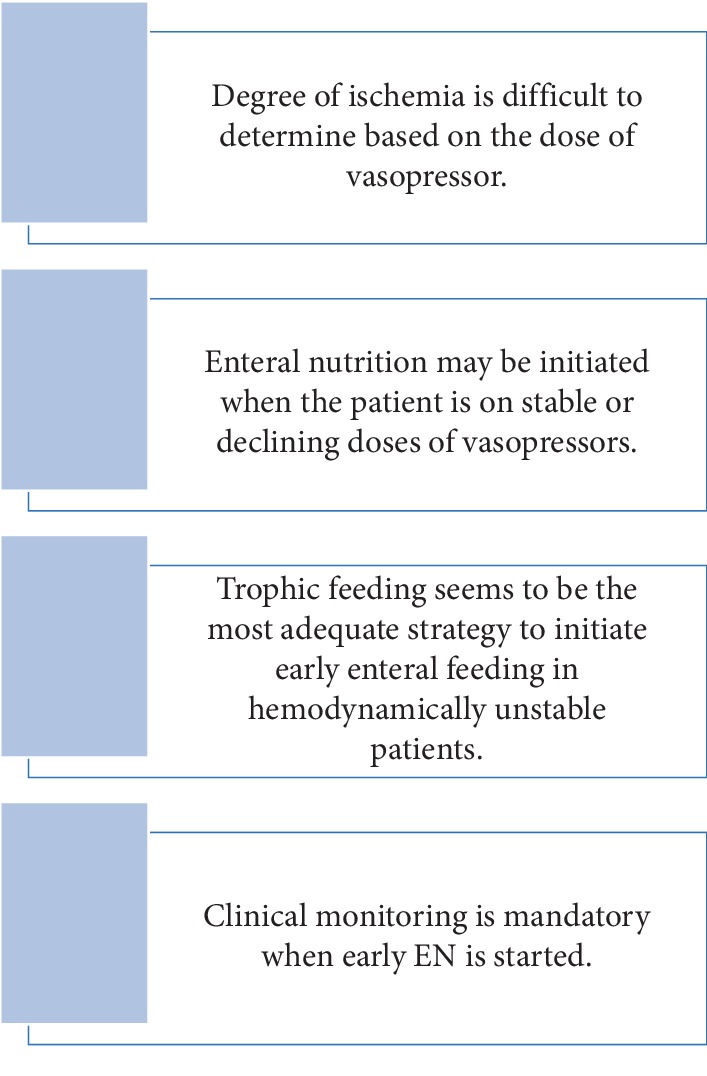
Take-home messages.

Figure 3 summarizes the take-home messages.

## 5. Conclusions

After reviewing the available literature, it has been observed that there are still many controversies about the supply of EN in critically ill patients using vasoactive drugs. Over the past few years, some myths have already been clarified. Nonetheless, there are still questions to be answered, especially regarding the safe dose of vasopressors, and it was not possible to identify a cutoff value for initiating this therapy.

However, current studies point to a higher rate of complications related to its introduction in patients with high doses or ascending doses of vasoactive drugs. The greater the complications, the higher the tissue hypoperfusion of the splanchnic territory, which is expressed in intolerance and the lower incidence, nonocclusive intestinal ischemia. According to current knowledge, despite drug doses, clinical signs are still the most important parameters in the evaluation of EN tolerance in the critically ill patient.

Therefore, we suggest that further studies should be conducted in this area, in order to guide the best clinical practice of ICU physicians, with the aim of identifying the safe dose of vasopressors to initiate EN in critically ill patients requiring vasoactive drugs.

## Figures and Tables

**Table 1:** Included articles and their main findings.

Author, year	Objective	Most common symptoms of EN intolerance (%)	Vasopressor drug used	Dose used	Mesenteric ischemia reported (%)	Main results
Reignier et al.	To investigate whether early ENT had beneficial clinical effects compared with early PN in patients requiring invasive mechanical ventilation and vasopressor support for shock	Vomiting (34%) and diarrhea (36%)	Adrenaline Dobutamine Noradrenaline	NR	Yes (2%)	In critically ill adults with shock, early isocaloric enteral nutrition did not reduce mortality or the risk of secondary infections but was associated with a greater risk of digestive complications compared with early isocaloric parenteral nutrition

Merchan et al.	To evaluate the tolerability of ENT in patients with septic shock who require vasopressor support and determine factors associated with tolerance of ENT	Gastric residuals >250 mL (74%)	Norepinephrine-equivalent	≤0.14 *μ*g/kg/min	No	Early EN may be tolerated and safely administered in patients with septic shock who are adequately fluid resuscitated and receive doses of <0.14 mg/kg/min of norepinephrine

Bruns et al.	To discuss the safe initiation of ENT concomitant with the use of vasopressors	NR	Norepinephrine Epinephrine Dobutamine Phenylephrine	NR	NR	Most postoperative patients requiring vasopressor therapy can likely be safely initiated and advanced on ENT. Administration of nutrition early in the course of critical illness is associated with improved outcomes and should be a primary goal in the treatment of these patients

Brisard et al.	To assess the hypothesis that early first-line ENT, as compared to early first-line PN, decreases day 28 all-cause mortality in patients receiving IMV and vasoactive drugs for shock	NR	Epinephrine Dobutamine Norepinephrine	NR	NR	In progress

Marik et al.	To provide an evidence-base assessment of factors leading to inadequate enteral nutrition support in critically ill patients	NR	NR	NR	NR	The benefits of early EN were greatest in the sickest patients and those receiving multiple vasopressor
Yang et al.	To summarize the effect of ENT and vasoactive agents on gastrointestinal blood flow and perfusion in critically ill patients, based on evidence	NR	NR	NR	NR	Current knowledge of EEN in critically ill patients with hemodynamic instability is still incomplete

Mancl et al.	To evaluate the tolerability and safety of ENT in critically ill patients receiving intravenous (IV) vasopressor therapy	Rising serum lactate (30.6%), elevated gastric residuals (14.5%), emesis (9.0%)	Norepinephrine	<12.5 *μ*g/min	Yes (0.9%)	EN is relatively well tolerated in patients receiving IV vasopressor support equivalent to 12.5 mcg/min of norepinephrine or less. Tolerability was less likely in patients receiving higher doses of IV vasopressors and in those receiving dopamine or vasopressin

Allen et al.	To review the effects of vasoactive substances such as pressors and inotropes on the gastrointestinal tract, as well as their use in combination with ENT	NR	Dopamine	3–10 *μ*g/g/kg/min	NR	The use of vasoactive substances should not entirely preclude clinicians from using the enteral route to supply nutrition. The evidence suggests that EN may be safely delivered to patients requiring vasoactive substances for hemodynamic support
			Dobutamine	12 *μ*g/kg/min or 200–800 mcg/min		
			Norepinephrine	6–25 mcg/min		
			Epinephrine	Doses not related to conclusion		
			Phenylephrine			
			Vasopressin			

Wells et al.	To review the effects of vasopressors on gastrointestinal blood flow, discuss complications associated with vasopressor use during ENT, and propose important considerations to determine the safety of ENT in hemodynamically unstable patients requiring vasopressor support	2 consecutive gastric aspirate volume (GAV) measurements between 150 and 500 mL, 1 GAV measurement	Dopamine	<5 *μ*g/kg/min	No	In the majority of ICU patients, administration of EN into the stomach during the provision of low, stable doses of pressors with close monitoring for signs of intolerance or worsening hemodynamic stability poses very little risk for bowel necrosis
			Epinephrine	0.3 *μ*g/kg/min		
			Norepinephrine	0.9 *μ*g/kg/min		
			Phenylephrine	0.5 *μ*g/kg/min		
			Vasopressin	—		

Khalid et al.	To determine the effect of early enteral feeding on the outcome of critically ill medical patients whose hemodynamic condition is unstable	NR	Norepinephrine, epinephrine, dopamine, or phenylephrine	NR	No	Early enteral nutrition may be associated with reduced intensive care unit and hospital mortality in patients whose hemodynamic condition is unstable

**Table 2:** Effect of vasopressors on digestive system circulation.

Agent	Receptors	Effect
Dobutamine	Beta 1	Increases cardiac output and blood flow to the mucosa
Dopamine	1 a 4 mcg/kg/min: dopaminergic receptors 5 a 10 mcg/kg/min: beta 1 11 a 20 mcg/kg/min: alpha	Increases cardiac output, redistributes flow to the serous
Norepinephrine	Alpha and beta 1	Increases splanchnic flow (up to certain doses)
Epinephrine	Alpha	Decreases splanchnic flow
Vasopressin	Direct vascular receptors	Generates intestinal vasoconstriction
